# Key factors influencing the professional development of golfers in South Africa: a reflexive thematic analysis

**DOI:** 10.1186/s13102-025-01239-7

**Published:** 2025-07-16

**Authors:** Stephanus Johannes Roos, Julius Jooste, Ankebé Kruger

**Affiliations:** 1 School of Management Sciences, North-West University, Vanderbijlpark, South Africa; 2 Psychology Department, Northumbria University, Newcastle-upon Tyne, UK; 3 Physical Activity, Sport and Recreation (PhASRec) / Centre for Health & Human Performance (CHHP), North-West University, Potchefstroom, South Africa

**Keywords:** Golf, Performance, Professional development, South Africa

## Abstract

**Background:**

An estimated 61.2 million adults play golf worldwide, with approximately 139,000 golfers affiliated with clubs in South Africa. Despite golf’s popularity as a recreational activity, the path to professional status and its sustainment is reserved for a few who demonstrate exceptional skill and dedication. Recognising the existing gap in research regarding the developmental trajectory of tour professional golfers in South Africa, there is an increasing need to identify the key factors that promote their professional development in this unique context. This study addressed this gap by aiming to elucidate the key factors contributing to their development.

**Methods:**

The study employed a qualitative descriptive design using semi-structured interviews with a purposeful sample (*N* = 17) of male and female tour professional golfers ranked within the top 100 on the Sunshine Tour and Professional Golfers Association of South Africa (PGASA) teaching professionals.

**Results:**

Reflexive thematic analysis identified nine overarching themes as key factors contributing to professional development in golf, namely a supportive developmental environment, competition-specific training, career management, a team of professional staff, financial resources, physical conditioning, lifestyle habits, psychological and physiological proficiency, and course management.

**Conclusion:**

This study’s findings may inform the development of a golf-specific framework for professional development tailored to a holistic approach in preparing golfers for professional advancement, highlighting its potential to optimise performance outcomes.

**Supplementary Information:**

The online version contains supplementary material available at 10.1186/s13102-025-01239-7.

## Introduction

Golf has become one of the most widely practised amateur sports globally [1]. The number of golfers in Africa has grown significantly since 2020, with 594,000 active golfers in 2022. Additionally, 5.3 million adults engage in other golf-related activities beyond traditional courses, such as driving ranges, par-three courses, and indoor simulators [2].

### Professional golf development in South Africa

In South Africa, junior golfers aged 5–18 compete in South Africa Kids Golf (SAKG) tournaments hosted in four provinces. The SAKG allows qualifying amateur golfers to participate in prestigious international tournaments and has been the pathway to success for several professional golfers [3]. The United States Kids Golf Foundation (USKGF) is another avenue junior golfers in South Africa use to learn to play golf in a competitive and fun manner [4]. Originally a golf equipment company, the USKGF has grown worldwide to provide players with a family-friendly environment to develop their golfing skills through competing in various international tournaments [4]. GolfRSA is the unified governing body for amateur golf in South Africa, overseeing both the South African Golf Association (SAGA) and Women’s Golf South Africa (WGSA). It manages 460 clubs and 139,000 members, promoting standardised governance, inclusive participation, and player development. GolfRSA maintains national handicapping and rules systems, organises competitions, and supports elite talent through structured training and National Squads [5]. Many amateur and professional golfers compete on the Bushveld Tour, a developing circuit that hosts two to three tournaments per month. It provides possibilities for emerging players to earn money, get competition exposure, and advance to higher-level circuits. Top performances in select events are also invited to the Big Easy Tour (supported by Ernie Els) [6], facilitating progression to international circuits such as the Sunshine Tour, South Africa’s premier professional golf circuit [7].

Amateur golfers are defined as players who compete in golf tournaments without being eligible to receive prize money [8]. Additionally, they are not allowed to accept compensation for coaching or permitted to be appointed as a PGASA teaching professional at a golf facility. In contrast, a tour professional golfer is a player who participates in competitions to earn an income and plays golf as a profession [9]. While many people play golf socially, only a few are good enough to play professionally [10]. In South Africa, tour professional golfers compete on the Sunshine Tour, where their primary income comes from tournament earnings rather than endorsements, allowing them to prioritise skills development due to fewer off-course commitments [11]. The Sunshine Tour prepares golfers for global success, as evidenced by the achievements of South African golfers worldwide [12]. The most successful professional golfers compete worldwide in the Professional Golfers Association (PGA) and the DP World Tour [11]. The PGA Tour has historically been the dominant governing body of golf, with professional golf tournaments mainly in North America [13]. A study by the Professional Athlete Index estimates that only 1 in 51,346 golfers in the USA reaches a professional level [14], and even then, success and a sustainable career in professional golf are not guaranteed [15]. The PGA Tour now competes with the LIV Golf League, a rival tour funded by Saudi Arabia’s Sovereign Wealth Fund. LIV has attracted top players with guaranteed contracts, fewer events, and substantially higher prize money, with a single tournament offering a purse of roughly US$4 million, and even awarding the last-place finisher a prize of $120,000 [13]. Players also benefit from lucrative endorsement deals [11]. Despite significant earnings potential, PGA Tour players face high costs. According to Ben Griffin, it costs at least $6,000 per week to compete [16], leaving non-winning players in financially precarious positions [17]. Some never record a win, while others may only win once over a 15-year career [18]. Consequently, professional golfers consistently seek out resources to support their development and sustain their careers [10], focusing on advancing their technical, mental [17– 19], physical [20], tactical [21], and life-skill capabilities [22, 23].

### Factors influencing golf performance

Olszewski-Kubilius, Subotnik and Worrell [24] state that effective career management is crucial for golfers, particularly through informed decision-making during key moments in their professional development. For golfers to make informed decisions, their professional team must comprise knowledgeable individuals, including different golf skill coaches, physical trainers, sports psychologists, physiotherapists, caddies, mentors, massage therapists, and managers/agents [17]. Regrettably, these professionals come with associated costs, and the availability of funds may pose a barrier for golfers in securing essential personnel to support them [25, 26]. Bliss and Langdown [27] assert that players should structure their training around technical and tactical skills and rounds on the golf course when planning their season. The technical aspects include putting, pitching, chipping, greenside bunker play, approach shots, and long game. Tactical and rounds of golf refer to strategies such as mapping the course for distances and the characteristics of the greens for tournaments, as well as time spent on the golf course preparing for competitive golf. In addition to the aforementioned, Bliss and Langdown [27] emphasise the importance of integrating physical conditioning such as strength, endurance, speed, mobility, and hypertrophy to complement rather than hinder technical and tactical development throughout the season. Moreover, enhancing physical conditioning increases clubhead speed [20] and reduces the risk of injuries [28], potentially leading to improved overall performance. Furthermore, golfers may experience less fatigue during and after the essential stages of a tournament [29].

To further enhance golfers’ performance, they must receive guidance from a coach knowledgeable in mental skills [10]. Players are required to perform the same technical skill under pressure in various circumstances [30] and a strong mental skillset is crucial for performance gains [18]. These mental skills typically include anxiety management [18, 31, 32], imagery [33, 34], attentional control [22, 35–37], pre-performance routines [38–40], and self-talk [22, 39, 41], as professional golfers often encounter pressure during tournaments, necessitating them to push their limits and challenge themselves during training sessions [19, 42]. According to Herder and Benoit [30], golfers strive to reach a stage where they achieve a “flow” state during competitive play. This “flow” state implies that players are not preoccupied with the outcome, such as their score or a technical aspect of their swing, but rather allow the swing to occur naturally. When flow is achieved, players often play a good shot and gain confidence and momentum to increase their performance [30]. Notwithstanding the abilities required to succeed in golf, non-ability variables in the immediate environment of golfers, such as pressure, prior experience, and scheduling, also influence their professional development [18]. In the South African context, Johnson-Brown [43] noted that limited resources, insufficient practice opportunities, and various external and internal pressures are frequently cited as factors hindering amateur golfers’ optimal performance. Although many golf academies support promising amateur golfers, uncertainties remain about their effectiveness in facilitating a successful transition [44]. Consequently, and based on the experiences of current professional golfers, Bliss [45] acknowledges that the literature lacks an inclusive and holistic framework for professional development.

### Theoretical underpinning in Understanding professional development in sport and golf

Numerous multi-stage frameworks exist for characterising athlete development. Notable examples include the stages of development framework by Bloom [46], the foundations, talent, elite, and mastery framework (FTEM) [47], the developmental model of sport participation (DMSP) [48], the role of deliberate practice in the acquisition of expert performance [42], long-term athlete development 2.1 (LTAD) [49], the differentiated model of giftedness and talent (DMGT) [50], psychological characteristics of developing excellence [51], the life-span model of the acquisition and retention of perceptual-motor expertise (LARPE) [52], and the athletic talent development environment (ATDE) model [53].

While these models underscore the role of various physical, psychological, and environmental factors in talent identification and development in sport, Gulbin and colleagues [47] point out several significant limitations. For instance, many existing models rely on a singular, generic pathway that is neither tailored to specific sports, such as golf, nor adequately supported by empirical evidence. Current models often isolate practice, overlooking the holistic factors influencing athlete development. Furthermore, these frameworks and models typically outline an athlete’s pathway chronologically, neglecting essential skill components and/or overemphasising psychological skills and individual athlete characteristics (e.g., Abbott & Collins’ model and the DMGT). This approach does not adequately consider the impact of environmental, economic, and social determinants, especially within developmental contexts such as South Africa.

Considering the noted limitations, Bronfenbrenner’s [54] social-ecological model of human development forms the foundation for this study, as it posits that human development is shaped by the dynamic interplay between personal and environmental factors in an individual’s embedded context. Rooted in community psychology, this model suggests that various social and physical factors influence an individual’s developmental experiences. As a result, development is determined by the interaction of processes, people, context, and time within a constantly evolving environment [55, 56]. Both Human [57] and Larsen, Alfermann, and Christensen [58] support the adoption of a social-ecological approach in sport, emphasising the need to recognise the wide range of influencers, such as coaches, support staff, parents, and peers, who contribute to an athlete’s development and retention in sport.

In the context of golf, Roos, Steyn, and Müller [10] set out to develop a framework towards professional development in golf within South Africa, which suggested that a holistic view of a golfer’s development is essential. In their proposed framework, these authors identified seven factors that may assist golfers in transitioning to becoming professional golfers. These factors include social support, coaching, specialisation, finance, psychology, lifestyle, and branding. However, Roos et al.’s study was limited by its focus on the perceptions of teaching professionals, excluding the views of active professional tour players in South Africa, a key stakeholder group in the development pathway. This constrained the analysis of factors critical to professional development, urging future research to incorporate a more representative sample that includes both active professional tour players and teaching professionals [10]. Additionally, the sample was predominantly male, with only one female participant, which may have restricted insights into the experiences and challenges female golfers face. Regarding the study design, a singular qualitative approach was used, which offered a limited exploration of the diverse and multifaceted factors crucial for sustaining performance at the professional level.

### The present study

Within the South African context, there is currently no well-defined method or framework exposing the pillars of successful development in golf. South Africa provides a unique landscape for studying golfers’ professional growth because of its inherent socioeconomic gaps, apartheid’s historical legacy, and the ensuing inequities in access to resources and athletic facilities. Furthermore, the country’s diversified cultural backdrop, climate variability, and geographic distribution of golf facilities result in developmental trajectories that differ significantly from those seen in more economically homogeneous or sport-specialised nations. These contextual elements demand locally grounded research to accurately reflect the influences affecting South African golfers’ professional trajectories. The evolution of professional golf involves several stakeholders, each with unique viewpoints and beliefs. Consequently, rather than depending on broad theories derived from more generalised athletic contexts, research should concentrate on isolating this community to examine collective perspectives on the matter.

Current theories of athlete development frequently highlight the importance of time progression and narrowly concentrate on individual abilities or traits, ignoring the interplay of many complex, multifaceted elements that impact development. In response to these limitations, the current study is essential for understanding the intricate dynamics and experiences that shape professional golf development. Guided by Bronfenbrenner’s ecological systems theory [55], this study explores the central research question: What factors contribute to the successful development of professional golfers in South Africa? Using this framework, the research offers a multi-layered understanding of the personal, interpersonal, and environmental influences on player development. The findings are intended to assist players better prepare for the demands of professional golf by emphasising the dynamic interplay between individual and contextual factors, both institutional and cultural. Finally, the study aims to inform a framework that promotes long-term career sustainability and allows golfers to realise their full potential in the competitive world of professional golf, particularly in South Africa.

## Methodology

### Study design

The study adopted a qualitative descriptive design, using individual semi-structured interviews to explore the views of South African tour professional golfers and the Professional Golfers Association of South Africa (PGASA) teaching professionals on professional development in golf. A qualitative descriptive design consists of analysing each aspect that influences the participant [59] and attempting to make sense of their feelings and thoughts [60]. To address the research question, the interpretation of semi-structured interviews was conducted in a constructivist and naturalistic manner, aligning with the social constructionist framework proposed by Crotty [61], which emphasises understanding participants’ lived experiences and personal perspectives. Interview schedule number 1 tailored specifically for tour professional golfers, was derived from a systematic review [62] investigating factors conducive to professional development in golf. Additionally, interview schedule number 2 was customised for PGASA teaching professionals. Utilising Bronfenbrenner’s ecological systems theory of human development as a framework, the standard questions and probes in both interview schedules ultimately sought to identify the contributing factors/influences within a professional golfer’s nested environmental systems, specifically the microsystem (immediate surroundings), mesosystem (interactions between microsystems), exosystem (indirect influences), macrosystem (cultural and societal context), and chronosystem (influence of time and historical events). With this said, it is important to note that the primary researcher (SJR) who conducted the interviews is an accredited PGASA teaching professional with nineteen years of coaching experience and a deep understanding of the golf context. Thus, the researcher’s deep understanding of the golf environment further ensured that the interview questions were not only relevant but also framed in a way that resonated with participants. This expertise helped in formulating probing questions that accurately captured the nuances of professional development in golf.

### Participants

This study included a non-probability sample (*N* = 17) of fifteen South African tour professional golfers and two PGASA teaching professionals, recruited using purposive and snowball sampling techniques. This method assisted the researcher in recruiting participants that may provide valuable information to the study [60]. The inclusion criteria required the professional golfers to be ranked among the top 100 players on the Sunshine Tour professional order of merit rankings and aged 18 years or older. The criteria set for teaching professionals required them to be full members of the PGASA and 18 years or older. In addressing the limitations identified by Roos, Steyn, and Muller [10], we argue that incorporating the perspectives of teaching professionals provides a more comprehensive understanding of the developmental process. These practitioners occupy a central role in talent development and offer critical insights into coaching environments, athlete progression, and broader structural influences, dimensions that may not be fully captured from the players’ perspectives alone.

The teaching professionals included in this study comprise a recognised PGASA award-winning coach, with both individuals having extensive experience coaching across all levels, from junior golfers to tour professionals. Table 1 below contains more information on the sample’s characteristics. No gender restrictions were set.

**Table 1:** Participant characteristics

Group	*N*	Gender	Age Range (Years)	Mean Age (Years)	Experience
Professional Golfers	15	8 Male 7 Female	23–41	Male: 26.25 Female: 24.57	4–22 years competing on a professional tour
PGA (SA) Teaching Professionals	2	1 Male 1 Female	22–55	–	37 years combined coaching experience (junior to tour level)

While the lived experiences of professional golfers are central to this study, the insights of two teaching professionals further enriched the data. Their perspectives, shaped by years of coaching and observing players’ development, provided a broader, longitudinal understanding of the factors influencing professional growth in golf. The interview process continued until no new patterns, or insights transpired from the interviews, indicating sufficient depth and redundancy in the data [60].

### Procedure

The Health Research Ethics Committee (HREC) of the Faculty of Health Sciences at the North-West University granted ethics clearance for the study (reference number: NWU-00176-22-A1). Data were collected through semi-structured interviews by the primary researcher of the study to understand the factors (negative and positive) that influence the development of professional golfers in the South African context. One pilot interview was conducted with a professional golfer to ensure the relevance of the interview questions and to evaluate the participant’s responses. Using a pilot interview, the adequacy of the research method was tested and ensured that data collection was completed correctly [63]. Based on the responses to the pilot interview, the researcher adapted the questions to ensure the study’s research question would be answered in the forthcoming interviews.

The PGASA served as the gatekeeper for the study. A designated PGASA employee was a mediator, distributing an information letter containing an overview of the nature and requirements of the study to PGASA coaches and their players. PGASA coaches are in contact with several professional players and acted as independent persons for the study. Upon completion of the study, a copy of the results and any publications emerging from the research will be made accessible to the participants to thank them for their time. Coaches first approached prospective participants to explain the research and facilitated communication with the researcher for any inquiries. Participants could then decide on their involvement in the study. If they chose to participate, the independent person provided the mediator’s contact details, which were subsequently shared with the researcher. The mediator functioned as a buffer between the researcher and participants, ensuring that the participants did not feel forced to participate in the study. Additional participants were recruited during a specific tournament and at practice facilities using snowball sampling. The interviews were completed in person at a time and place most convenient for the participants and took between 20 and 40 min. During the interviews, informed consent and a demographic sheet were conferred. Furthermore, the participants were reminded that no personal information would be made available to the public and that their anonymity and confidentiality would be ensured. Furthermore, participants were informed that they could withdraw from the study at any time before formal data analyses without facing any consequences.

### Data analysis

The data analysis was done using Braun and Clarke’s method of reflexive thematic analysis, which is known for its theoretical adaptability in facilitating the exploration, examination, and interpretation of patterns within qualitative datasets [64]. The study followed a deductive approach to assess how the professional development of golfers aligns with Bronfenbrenner [54] social-ecological model. Additionally, an inductive approach was used to explore additional factors that might influence the professional development of golfers. Integrating these approaches facilitated a thorough understanding of the phenomenon [60]. The audio recordings of the interviews were transcribed verbatim, imported into the qualitative data analysis software Atlas Ti, and used for analysis. According to Braun and Clarke [65], reflexive thematic analysis contains the following steps:

(i) **Familiarisation with the dataset**: This initial phase entailed the primary researcher reading the data numerous times to become immersed and familiar with its contents. Detailed notes were made to record analytical observations concerning the interview transcripts and the audio recordings.
(ii) **Coding**: In this phase, codes were generated to capture critical features of the data pertinent to addressing the research question. The entire dataset is systematically coded during multiple rounds of coding.
(iii) **Generating initial themes**: This phase involved examining the codes and collating data to identify overarching patterns of meaning. Data relevant to each potential theme were grouped together to aid in further exploration and evaluation of their validity.
(iv) **Developing and reviewing themes**: In this phase, the themes were evaluated by the researchers against the coded data to ensure they formed a coherent narrative reflection of the data and aligned with the research question. Following this, the themes were further refined, including splitting and combining specific themes and discarding irrelevant themes.
(v) **Refining**,** defining**,** and naming themes**: This phase entailed a comprehensive analysis of each theme, determining its scope, focus, and narrative both deductively and inductively aligned to Brofenbrenner’s ecological model. Additionally, this stage entailed determining a descriptive and informative name for each theme. Furthermore, an independent researcher reviewed the key themes to verify their representation of the entire dataset and to examine the quotes supporting each theme for inclusion in the report.
(vi) **Writing up**: The final phase involves synthesising the analytical narrative and pertinent data extracts, situating the analysis in the broader context of existing literature.

### Methodological rigour

Qualitative researchers’ subjective perspectives, or biases, are intricately linked with the research process [66]. While it is useful for the researcher’s perspective to guide the research process, failing to use trustworthiness methods such as dependability, confirmability, and transferability of the data will negatively affect the study [60, 67–69]. Therefore, after transcribing each interview, the transcripts were returned to the participants for member checking. This process confirmed the accuracy of the transcriptions and allowed participants to contribute any additional perspectives that may have been left out [60, 69]. To further strengthen the credibility and validity of the research study, efforts were made to identify cases that challenge and contradict the emerging results and concepts, offering the researchers involved deeper insights into the subject matter [60].

Throughout the data collecting and analysis phase, the researcher used ongoing reflexivity to improve methodological rigour. This required keeping a reflective notebook in order to assess potential prejudices, presumptions, and biases that might affect how data is interpreted. Regular conversations with supervisors and peers also made it possible to guarantee that the constructed themes were based on participant narratives rather than the researcher’s assumptions or past knowledge. The researcher reduced the possibility of subjective influence and promoted transparency by actively challenging their position in directing the research process, which increased the findings’ legitimacy and dependability [60]. Furthermore, the study included comprehensive descriptions of the research setting, participants, and their responses to support each identified theme [60]. As mentioned previously, the primary researcher (SJR) who conducted the interviews is an accredited PGASA teaching professional with nineteen years of coaching experience and a deep understanding of the golf context. This expertise also helped establish rapport with the participants, reducing response bias and encouraging detailed, authentic reflections on professional development. The interviewer/primary researcher’s deep understanding of golf’s technical, psychological, and developmental aspects allowed for more precise probing, capturing nuances that a non-expert interviewer might overlook. Furthermore, his ability to interpret golf-specific terminology and contextual challenges ensured the accuracy and depth of the data, ultimately enhancing the study’s validity. Additionally, fellow researchers (JJ & AK) are registered practitioner psychologists and academics with extensive experience analysing and interpreting qualitative data, further strengthening the study’s methodological rigour.

### Results and discussion

The research question *What factors contribute to the successful development of professional golfers in South Africa?* was investigated using reflexive thematic analysis of the interview transcripts. Through an iterative and reflexive process of coding and analysis, nine themes were generated, representing patterns of meaning constructed from the data: ‘Supportive developmental environment’, ‘Competition-specific training’, ‘Career management’, ‘Team of professional staff’, ‘Financial resources’, ‘Physical conditioning’, ‘Lifestyle habits’, ‘Psychological proficiency and talent’ and ‘Course management’. The themes of ‘Supportive developmental environment’, ‘Career management’, ‘Team of professional staff’, ‘Physical conditioning’, ‘Lifestyle habits’, and ‘Mental skills’ were confirmed as key aspects identified in the ecological model by Bronfenbrenner [54] and directly assist in answering the research question. The remaining themes enrich the data by providing additional insights into ‘Competition-specific training’, ‘Financial resources’, and ‘Course management’. While our study primarily focused on the mechanisms and influences shaping professional development trajectories for those who have already entered the sport, we did not include access to golf as an analytical focus. However, we did identify financial capacity, which is closely tied to access, as a pervasive and critical determinant of progression and success in professional golf. In line with Bronfenbrenner’s paradigm, financial obstacles exist at many ecological levels. Costs associated with coaching, equipment, and tournament travel have a direct impact on individual developmental experiences at the micro and meso levels. Furthermore, systemic disparities, institutional regulations, and socio-historical legacies influence who has access to opportunities in the sport at both the exosystem and macrosystem levels. Therefore, access was not the central research question, but it should be noted that it remains an underlying structural factor that permeates the entire developmental system in golf.

#### Developmental environment

Participants emphasised that the early stages of golf development should prioritise a supportive environment. Furthermore, many players emphasised the critical role of employing competent coaches in fostering their professional advancement. In addition, participants indicated that for golfers to continue developing, they must enjoy the developmental process and be exposed to a pressure-free social support structure conducive to sufficient growth. A summary of the theme representing a golfer’s developmental environment is indicated in Table 2.

**Table 2:** Summary of the theme representing a golfer’s *Developmental environment*

Theme	Sub-themes	Theme description	Sample response
Supportive developmental environment	Strong support system	A network of supportive individuals offering emotional, financial, and practical assistance in a non-pressurised environment.	*“…but my parents were very supportive…” (P6)* *“100%. A lot of good support…But yeah*,* my support system*,* my family*,* my wife. We’ve had*,* yeah*,* I’ve got a very good support system. Very*,* very lucky with that.” (P15)*
	The coach	Knowledgeable coaches for technical refinement and holistic athlete development, while recognising the importance of avoiding early sport specialisation.	*“Probably the biggest impact was my current coach. He is super knowledgeable*,* and been around for quite long*,* and has coached lots of guys at high levels.” (P2)* *“And I think the new set of eyes has just given me a different perspective on how to do the things I needed to do.” (P12)*
	Delayed specialisation and extensive sports participation	Engage in multiple sports to develop a broad range of skills and only specialise in one sport at around 15 years old.	*“Also*,* if they are young*,* they just need the basics*,* you know*,* the grip*,* the stance*,* everything. From the age of*,* let’s say*,* 12 to 15*,* that’s when you need to be a bit more technical for them.” (P10)* *“Yes*,* yeah. Yeah. Lots of other sports*,* pretty much anything. Because it was all fun as well. Tennis*,* squash*,* and swimming.” (P2)*
	Enjoyment	Early stages of golf development should consist of a supportive and enjoyable environment.	*“Because golf should be fun.” (P5)* *“You want to just be out there playing the game you love and having fun.” (P15)*

##### Strong support system

Several participants emphasised the significant influence of social support on their performance and its critical role in achieving success. None reported experiencing undue pressure from their parents to excel in golf. However, some noted that external stressors significantly influence their performance. Many participants highlighted the importance of encouragement from a supportive group sharing their goals. Participants specified that players’ support structures significantly affect their success on the golf course and that an essential factor in their success was that they never experienced undue pressure from their parents.

Interviews with participants affirm the findings of Hayman, Borkoles, Taylor, Hemmings and Polman [70], indicating that golfers’ developmental environments are significantly shaped by their social support networks, particularly parental involvement. Participants underlined the importance of a supportive and pressure-free atmosphere during their early engagement with golf [10, 70]. Fathers are notably influential [71], providing coaching, financial, and emotional support in the initial stages but transitioning to a more supportive role around ages 14–16, with coaching typically shifting to qualified instructors [70, 72]. Similarly, Swedish golfers have indicated that their parents or close friends of their parents were significant drivers in the early stages of their golf participation [73]. For golfers to continue their development, they need coaches who encourage an enjoyable and pressure-free environment to develop in [10, 70].

##### The coach

Participants indicated that the appropriate coach for each player is crucial for professional development. Furthermore, participants stressed that coaches must work with golfers’ natural abilities and that golfers should be aware of coaches who may lead them in the wrong direction.

*Coaching is very important. (Participant 13)*

*I’m not teaching a putting stroke. You saw that I don’t do the putting stroke; I just put the structures together to have the best stroke that you can have*,* which is what you do naturally. (Participant 5)*

*Definitely having the right people around you*,* the right coaches*,* giving you the right information and not misleading you. (Participant 3)*

Hayman, Borkoles, Taylor, Hemmings and Polman [70] support the notion by participants that when golfers commit seriously to their professional development, there is a need for a well-qualified coach to refine the technical aspects of their armoury. Therefore, participants indicated golfers’ need for knowledgeable coaches to assist them in their professional development [74]. Moreover, coaches are tasked with the holistic development of athletes, encompassing various dimensions such as technical, physical, and psychological areas [10, 74].

##### Delayed specialisation and extensive sports participation

All participants viewed delayed specialisation as important, with most participants indicating that they participated in various sporting codes from a young age and only specialised in golf at approximately 12–15 years old.

*Also*,* if they are young*,* they just need the basics*,* you know*,* the grip*,* the stance*,* everything. From the age of*,* let’s say*,* 12 to 15*,* that’s when you need to be a bit more technical for them. (Participant 10)*

*Yes*,* yeah. Yeah. Lots of other sports*,* pretty much anything. Because it was all fun as well. Tennis*,* squash*,* and swimming. (Participant 2)*

Consistent with the views of the participants, Swedish golfers also tend to delay specialisation in golf, which typically occurred between the ages of 15 and 20 [73]. Players are encouraged to participate in as many sporting codes as possible at a young age and only specialise in a specific sport at a later stage [10, 70]. According to Hayman, Borkoles, Taylor, Hemmings and Polman [70], exposure to multiple sports during the developmental years, rather than early specialisation, promotes the development of a range of cognitive, physical, and social skills essential for a successful golf career.

##### Enjoyment

Participants highlighted that playing golf was always enjoyable and formed an essential part of their journey.

*So yeah*,* I sort of started playing junior tournaments*,* as well*,* just for the love of the game*,* sort of enjoying that. (Participant 11)*

The study by Hayman, Borkoles, Taylor, Hemmings and Polman [70] and Hayman, Polman, Taylor, Hemmings and Borkoles [72] indicated that the early stages of golf development should create an enjoyable environment, as highlighted by the participants of this study. Roos et al. [10] also believe that golfers must enjoy playing golf to sustain their participation and achieve successful development; otherwise, discontinuation is likely to occur.

#### Competition-specific training

The second theme from the semi-structured interviews was competition-specific training, which participants highlight as crucial for professional golfers to prepare adequately for competitive environments. The participants indicated they needed to be challenged during practice sessions to emulate the competitive environment they would encounter during tournament play. They noted that everyone is different and should have a routine that works best for them. Furthermore, according to the participants’ responses, competition-specific training should encompass elements such as variability and deliberate practice, essential for their performance in competitive settings. A summary of the theme representing the importance of competition-specific training is indicated in Table 3.

**Table 3:** Summary of the theme representing the importance of *Competition-specific training*

Theme	Sub-themes	Theme description	Sample response
Competition-specific training	Coaching techniques– variability and deliberate practice	Training should challenge the golfer and simulate tournament conditions while conserving energy and emphasising the short game through varied practice scenarios.	*“So*,* every single shot you are hitting was a different shot*,* kind of a different lie with a different club. So*,* it’s never the same shot over and over again.” (P4)*
	Technology usage during training	Tools such as Trackman and Flightscope should be used for drills, but not to the extent that they impede the golfer’s intuitive feel for the game.	*“I have my own Trackman. I use it for dialling in wedge distances especially.” (P3)*
	Custom fitting and correct equipment	Equipment must be tailored to match the golfer’s natural swing.	*“…what you need to do is get the club built to your swing and what shape you want to hit.” (P15)*

##### Coaching techniques: variability and deliberate practice

Participants described a two-stage approach to tournament preparation involving a “play box” and a “drill/practice box”. The “drill/practice box” refers to the time golfers spend on the driving range practising specific techniques and drills, while the “play box” involves players concentrating on the centredness of strike and speed, with minimal emphasis on technical details. One participant noted that she applies this technique with her clients. Another participant mentioned beginning preparations three days before the tournament by playing 18 holes, followed by two rounds of nine holes until feeling comfortable. Additionally, another participant highlighted the importance of incorporating various games into practice sessions to challenge herself.

*Yeah*,* like preparing for events is there. Just to say*,* there’s a difference between a practice box and a play box*,* as he calls it. So*,* when you’re practising*,* obviously approach it a certain way. Think about how you want to swing*,* what you’re working on*,* and things. And then in the tournament*,* then you switch to the play box*,* and then you forget kind of as much as you can from the practice box. (Participant 2)*

*So*,* I feel like kids need to take what I’ve taught them. They take their playbox and drill box and need to take that on the course. And practice.” So*,* I have the first one*,* which is a drill box. So that’s your box. I have five golf balls. They focus on*,* for example*,* takeaway and press on their left foot. So*,* they practice that with their drills. When they have a playbox within their mind*,* they still need to feel the takeaway and the pressure*,* but they need to focus on centre strike or speed. Then*,* when we get on the golf course*,* I play with two balls. So*,* the first ball is your drill. The second ball is your play box. (Participant 10)*

One participant highlighted the importance of purposeful practice using different lies and clubs, while another stressed the necessity of structured practice for golfers. Additionally, a participant noted that preparation should be tailored to specific needs, emphasising the importance of conserving energy.

*So that’s what I see with him*,* very structured: gym*,* eat properly*,* practice from this time to this time*,* practice my putting*,* do this*,* go and practice bunkers*,* work on my Trackman. He’s got a structure like a very work-like structure to do what he needs to do to be as efficient as he can. (Participant 5)*

*So*,* it depends from week to week. Some weeks*,* I swing the thing well*,* and then I don’t have to*,* and then I just play. Especially in Asia*,* there is more of an energy reserve build-up because*,* again*,* 40 degrees every day*,* you spend a minimum of 6 to 8 h out there*,* and you still have to train before and after the time for four days and you have just flown 20 h to get there. (Participant 14)*

The participants’ responses are in agreement with Hayman, Polman, Taylor, Hemmings and Borkoles [72] that deliberate practice is crucial for developing golfers committed to professional growth. This systematic, goal-oriented training enhances both physical and mental performance [75] and becomes essential when golfers focus on a long-term professional career. When this focus occurs, golfers must make use of a professional golf coach and realise the importance of critically analysing their game and strategically planning their practices [70]. According to Highfield, Cooke, Zeigel and Parker [19], coaches must incorporate spacing, variability and a challenge point into their training sessions. This training method involves regular changes in both the club selection and target and the incorporation of challenges designed to heighten the demands placed on golfers, as may be experienced in a tournament environment [19]. When golfers are unable to replicate the tournament environment successfully, they can use launch monitors like Trackman to compete on virtual golf courses or undertake challenges offered by the software [76]. Furthermore, Rittenberg, Barnhart, Neyedli, Young and Dithurbide [77] found that golfers are more likely to use technology when their coach encourages them, further stressing the importance of coaching techniques as a vital factor contributing to a golfer’s professional development.

##### Technology usage during training

Many participants rely on technology during practice sessions, using video analysis and launch monitors, such as Trackman and Flightscope, as an essential part of their preparation. Furthermore, according to the participants, these devices are primarily used for distance control with approach shots. Certain participants indicated that when they rely too much on technology usage, they have limited feel when executing short game shots and become too technical.

However, one golfer expressed that relying on data such as clubhead speed and swing path made her approach overly technical, preventing her from achieving her desired scores. Another golfer mentioned infrequent use of launch monitors during practice sessions, while a third player found Trackman to be confusing. Additionally, one golfer indicated a desire to use Trackman but cited its high cost as a barrier.

*Yes*,* I used to*,* but I lost feel when using it like I forgot how to score. (Participant 1)*

*Like you pay five times what you would for a normal bucket of balls to hit off Trackman. I can’t pay R300 a day just to hit on Trackman. (Participant 8)*

*I use it a bit*,* but not much. The numbers confused me a bit. (Participant 9)*

Conversely, some golfers emphasised the importance of launch monitors for distance control, viewing them as essential tools. One golfer noted that Trackman is crucial for her drills, while another reported using it with junior players during practice sessions to facilitate games.

*I’m saying physical drills*,* things that are on my body in the ground. In my eyeline*,* we use a lot of those*,* and then also Trackman. (Participant 7)*

*So*,* like*,* Trackman has games. Now*,* we do their tipping game with them in a pitching game. So*,* they have a distance from 24 to 50*,* for example*,* and they play their game even though their score is not high*,* they still have their fun opportunity to play on the machine*,* you know. (Participant 10)*

Corresponding with the participants’ feedback, athletes’ use of technology has increased significantly in recent years and has become essential for gaining a competitive advantage [77]. Measurement technologies like launch monitors offer golfers immediate feedback on critical metrics such as ball speed, clubhead speed and angle of attack [78, 79]. Golfers are encouraged to use tools like Trackman and FlightScope cautiously to align with their needs, as over-reliance on technology can diminish their natural abilities [77]. This technology allows golfers to enhance their performance by identifying areas for improvement and optimising training protocols [80]. Furthermore, players must adapt to various golf courses and environmental conditions using their own equipment [76].

##### The importance of custom fitting and correct equipment

Participants (15, 16 and 17) note the importance of the correct equipment matching their swing characteristics.

*Yeah*,* it’s definitely a big*,* big thing. I think having the wrong equipment can cause you to start swinging it differently. And then you start to look at the golf swing when that wasn’t a problem in the first case*,* you know*,* so*,* yeah*,* it’s*,* it’s big to know that your equipment is suited to your game*,* and you can go and swing it the way you are capable of and not have to worry about that. (Participant 17)*

Supporting the participants’ feedback, Haeufle, Worobets, Wright, Haeufle and Stefanyshyn [81] also emphasised that golfers should be familiar with their equipment and ensure it is built and adjusted to their specific swing characteristics. Furthermore, flexibility [82], length [83], kick points [84] and weight [81, 83] of the shaft in the golf club has a significant influence on a golfer’s game [85]. Consequently, golfers must have the appropriate head and shaft to attain the intended ball flight. To accurately determine the correct equipment an individual needs, they are encouraged to complete a custom fitting with an equipment specialist [81]. The game of golf has evolved to the extent that professional golfers leverage experts in diverse fields to join their teams to gain a competitive edge, such as equipment specialists, coaches, strength and conditioning specialists, and sports psychologists [17].

#### Team of professional staff

The third theme derived from the data was diversified team composition, emphasising a support team’s role in facilitating professional golfers’ development. Each professional golfer who was interviewed indicated that they have a team of people around them assisting in enhancing their performance. The team of individuals included a short-game and long-game coach, a physical trainer, a sports psychologist, a physiotherapist, a caddie, a mentor, a chiropractor, a massage therapist, and a manager. A summary of the theme representing the importance of having a Team of professional staff is indicated in Table 4.

**Table 4:** Summary of the theme representing the importance of having a *Team of professional staff*

Theme	Sub-themes	Theme description	Sample response
Team of professional staff	Short and long-game coaches	Each aspect of– golf, chipping, putting, and long game– requires its own specialised coach.	*“I got a coach*,* long game and short game*,* so I’ve got a putting coach and a long game coach” (P7)*
	Mentor	Experienced professionals can act as valuable mentors to offer essential insights into the culture and systems of professional golf.	*“I think any successful person has a mentor*,* someone that you can always phone or ask or just guides you in the right direction.” (P3)*
	Caddie	An experienced caddie is essential to guide players in high-pressure situations. The significance of trust, psychological support, and communication skills are vital for optimising performance.	*“…your number one investment is getting yourself a good caddie because if you get yourself a good caddy*,* and he can save you one shot around*,* he’s really paid off threefold.” (P7)*
	Golf psychologist	A golf psychologist supports mental health, assists in managing pressure situations, and ensures the longevity of players.	*“…someone that can help you do mental side of things*,* and sort of using them to the*,* to get the most out of each day.” (P11)*
	Physical trainer /Physiotherapist	Physical trainers and physiotherapists focus on fitness, mobility, and strength in players.	*“I have*,* I don’t always listen to him*,* but I have some.”… “For me*,* it’s about flexibility*,* mobility…” (P14)* *“…and into biokinetics” (P2)*
	Manager/agent	Involves the logistical aspects of a golfer’s life beyond the on-course activities of players.	*“…that’s the main thing about having a manager is that he’s just trying to make your life a little bit easier.” (P3)* *“It was just kind of my agent.” (P3)*

##### Short and long-game coaches

Several participants underscored the significance of the short game in their success and highlighted employing various types of coaches, including those specialising in both short and long-game techniques. One golfer emphasised the crucial roles of her putting and long-game coaches, while another highlighted her team’s inclusion of a putting coach and a short-game coach. This underscores participants’ collective view of the importance of both short- and long-game coaching, particularly the need for exceptional skills in putting and chipping.

*A golf coach is somebody who helps with my putting and short game. (Participant 3)*

*So*,* I wish that somebody when I was younger made me focus more on the shorter short game and the putting*,* which only later in my career did I realise how much that actually matters. (Participant 7)*

*Then I go to practice chipping and putting. That’s where the game is played. (Participant 13)*

As noted by various participants, professional golfers often engage in various specialised coaches to address different aspects of their game. These may include coaches focusing on putting, short game, long game, and mental conditioning, each providing targeted expertise to enhance the golfer’s overall performance [17]. Golfers in a study conducted by Hayman, Borkoles, Taylor, Hemmings and Polman [70], also benefited from mentoring relationships with tour professionals early in their careers, gaining valuable insights.

##### Mentor

Golfers derived substantial benefits from the tacit knowledge imparted by mentors regarding the culture and systems of high-performance golf. Participants emphasised the significance of having a supportive figure, such as a mentor, to rely on and engage in discussions, which they found instrumental in achieving success.

*One of the biggest things*,* you have to have a good mentor. That’s number one*,* you have to have a good mentor*,* someone you can rely on. (Participant 16)*

*He’s also played on tour before*,* and he knows the ins and outs. (Participant 3)*

Reinforcing the participants’ responses, various researchers [24, 86, 87] found that having an experienced mentor enables athletes to learn about navigating sports systems, whom to cultivate, and what pitfalls to avoid during their developmental process. In this respect, Hayman, Borkoles, Taylor, Hemmings and Polman [70] noted that fathers frequently served as mentors for golfers, exerting a considerable influence on their developmental trajectory.

##### Caddie

Numerous participants asserted the importance of a proficient caddie for success, particularly in high-pressure scenarios. Additionally, several professionals in the field have preferred caddies capable of engaging in non-golf-related conversations, facilitating diversion from the immediate focus.

*Very*,* very*,* very important. They can help you bring up what you’re really capable of on the golf course. You know*,* I think a caddy can interfere with that. And get in the way of things if he’s if he’s not the right fit for you. But having the right caddy can really help you. Just go out there and play golf; you don’t have to think about it too much. (Participant 17)*

*When you’re under pressure*,* can they bring you back down? Can they calm me down? Can they keep you in the moment? Because it doesn’t matter what you say? There’s nobody that can do that themselves. I mean*,* like*,* I know well if I’m under the gun and I’m leading by one shot or on one shot behind? How are you going to control yourself? (Participant 7)*

One golfer indicated that using a caddie is crucial for inexperienced golfers to help them avoid mistakes early in their careers, while another golfer noted that having a trusted caddie is essential. Furthermore, participants emphasised that the “mood” and the non-golf-related discussions a caddie has with the golfer are crucial. Additionally, participants observed that sometimes, a non-golfer who can distract a golfer’s mind from the situation is very effective.

*It was necessary for me to find someone who was going to help me stay calm and to just stay calm on the golf course more than I needed someone to tell me exactly what to do. (Participant 13)*

*For me*,* it’s important to have someone whom I can talk to*,* watching everyone. They say you need a caddy who is good and so on*,* but you actually need a caddy who is good to talk about*,* not golf either. (Participant 16)*

*And me and him work really well together*,* and I trusted him. So*,* when he made a comment*,* and he wanted me to hit a different club or do something else*,* I did that with confidence because I trusted in his ability as a caddy. (Participant 4)*

Various researchers concur with the prevailing view that the advantages provided by a proficient caddie are significant [88–90]. Donald and Winter [88] and Pilgrim, Robertson and Kremer [90] agree that caddies have a much more significant role than just the practical aspects, such as carrying a bag and cleaning clubs; they also play an important role in the psychological conditioning of golfers. Golfers aim to achieve an ideal psychological state characterised by seamless performance, self-confidence, focus, relaxation, present-moment awareness, and the ability to mentally reset between shots [90]. Caddies frequently aid in this process through non-golf-related conversations, post-shot reflection and positive reinforcement using key phrases such as “good shot” or “great swing” [88]. Moreover, caddies provide golfers with crucial information regarding the required shot for a particular situation. Furthermore, caddies must be consistent in their behaviour and all tasks during tournament situations [88]. Trusting your caddie is vital for professional golfers as they form a reliable source aiding in decision making under pressure [90]. This decision making includes club selection, which reduces a golfer’s doubt and increases self-confidence before shot execution [90]. The caddie often analyses the psychological state and facilitates the player’s psychological performance accordingly [88], often in conjunction with the support of a golf psychologist.

##### Golf psychologist

Many participants highlighted the importance of robust psychological skills, noting that although some players do not utilise a golf psychologist, such support aids them during pressure situations. One golfer mentioned that his team includes a mental trainer. Another emphasised the importance of having a sports psychologist, noting that this support contributes to her longevity in the sport.

*And just health-wise*,* for your longevity? I think that plays a huge part to know you have someone to talk to all the time. (Participant 8)*

*I have a sports psychologist. (Participant 9)*

Hayman, Borkoles, Taylor, Hemmings and Polman [70] emphasises that developing robust psychological skills such as perseverance [91, 92], confidence [22, 93, 94], anxiety management [18, 22, 32, 93], acceptance after a bad shot [38, 95], attentional control [22, 35–37, 96, 97], imagery [33, 34, 98] and self-talk [22, 39, 41, 99] are crucial for golfers striving for professional success. Hickman and Metz [100] highlight that golfers are often under intense pressure during tournaments. Consequently, golfers must achieve an appropriate psychological state to effectively execute the intricate technical skills required for specific shots [101]. Smith [102] underscores that success in golf hinges on a combination of technical, physical, tactical, mental, and life-skill capabilities. According to Carson [103], professional golfers rely on physical trainers to enhance their athletic performance.

##### Physical trainer/physiotherapist

Participants underscored the significance of physical conditioning in enhancing their performance, highlighting the substantial benefits of a dedicated physical trainer. They noted that physical trainers play a pivotal role in optimising their physical fitness, which is essential for achieving peak performance in golf. One participant emphasised the importance of having a physical trainer and physiotherapist for mobility and flexibility, while another highlighted a support system that includes a coach, mentor, caddie, and supportive family and friends, along with a physical trainer.

*And then obviously my physio and my trainer*,* physical trainer. (Participant 3)*

*So*,* I obviously have my coach as well as a physiotherapist and a biokineticist*,* and that’s about all the people I work with in the golf industry. (Participant 6)*

Yoon and colleagues’ [104] findings align with the prevailing view of the participants, which indicates that developing robust strength and conditioning levels positively contributes to golfers’ overall performance. These physical attributes include hip mobility, core control, thoracic mobility, hip hinge ability, lower body strength and pelvic tilt ability [105]. Furthermore, physical conditioning directly correlates with increased clubhead speed among golfers [20]. These findings emphasise the importance of engaging specialists such as trainers to enhance physical conditioning, improving various aspects of a golfer’s ability. In addition, athletes often employ managers and/or agents to manage their brand [106].

##### Manager/agent

Participants highlighted the valuable role of managers/agents in simplifying players’ lives, enabling them to concentrate solely on their golf performance. Some golfers consider having a manager/agent essential, particularly for assistance with travel logistics when competing abroad.

*I’m talking to a management company on Thursday who might be helping me because it’s also necessary*,* it helps*,* it takes the pressure off you. (Participant 13)*

In contrast, one golfer who does not have a manager handles all logistical bookings independently.

*I don’t have a manager. I don’t think it’s terribly difficult to book plane tickets and hotels these days; you do it all on an app. Why should I pay a guy to do it. (Participant 14)*

The individual who supports professional golfers with off-course responsibilities is typically referred to as a manager or agent [106, 107]. These professionals manage multiple aspects of a golfer’s career, including securing sponsorship deals, exploring endorsement opportunities, and developing comprehensive post-career strategies [108]. In line with the participants’ interview responses, Gordin [17] states that professional golfers competing on the PGA Tour typically recruit the services of a manager or agent to support their career pursuits.

#### Career management

The fourth theme that was constructed from the data was career management, emphasising the crucial effect of decision-making and accumulated experience in fostering the development of professional golfers. Participants indicated that the decisions made during professional golfers’ career management are essential. Many professionals suggested they could have achieved success earlier in their careers had they approached their decision-making processes differently. A summary of the theme representing the importance of career management is indicated in Table 5.

**Table 5:** Summary of the theme representing the importance of *Career management*

Theme	Sub-themes	Theme description	Sample response
Career management	Decision making	The decision-making regarding tournament scheduling and burn-out in players is crucial.	*“But I think decision making when it comes time to know*,* you know*,* turning pro*,* deciding on the team around you*,* deciding on which queue schools are gonna go to what opportunities you’re going to take. I think decision-making is huge.” (P17)* *“So*,* it was bad decision-making. But I was too young to know the difference.” (P1)*
	Developmental opportunities and experience	Addressing the shortage of tournaments and lack of sufficient resources, particularly for female professional golfers.	*“We started losing events. We went from 28 to nine. And I’m not going; I can’t make a living playing nine tournaments. That’s nine weeks. What do you do for the other?” (P1)* *“…experience and years out here teach you what you need…” (P14)*

##### Decision making

Various decision-making processes for scheduling golfers’ events were indicated as essential and have affected their development. Several professional golfers said that when decision making is not done well, they cannot perform at their best, which may lead to burnout. Discussions highlighted how ineffective decision-making hindered successful transition to professional status, suggesting that adequate career management could enhance success. Poor scheduling decisions were noted as detrimental to development, while participants acknowledged that excessive tournament commitments, resulting from poor decision-making, can lead to burnout.

*I played terribly at Q school like I had burned out by the time I got there*,* you know*,* I was playing trying to play way too many events. (Participant 12)*

*I was just tired. Scheduling is very important. And you shouldn’t be afraid to say no to tournaments. (Participant 14)*

Lee [23] and Roos, Steyn, and Müller [10] agree that golfers frequently drop out of the sport due to burnout. Burnout is particularly prevalent among junior golfers, who often participate in too many tournaments under pressure from their parents [109]. Furthermore, parents frequently make ill-advised decisions regarding the timing of specialisation in golf and the selection of coaches for their children, which can significantly impede the golfers’ development. Professional golfers can benefit greatly from mentorship by individuals who have a thorough understanding of the professional culture in golf, which can help mitigate poor decision-making [24]. As such, guidance during the professional development of golfers is essential for them to experience and succeed at an elite level [72].

##### Developmental opportunities and experience

The interviews revealed that female professional golfers lack sufficient tournaments, experience, and resources. There appears to be a noticeable disparity in the support female professional golfers receive compared to their male counterparts. Despite considerable talent among female professional golfers in South Africa, there is a dire need for more opportunities to enhance their skills to help them reach the highest level in golf. Participants underscored the limited availability of tournaments and opportunities for female professional golfers in South Africa, which hinders their professional development. Many emphasised that experience is vital for reaching a professional level, with one participant noting that his college golf experience in the USA greatly benefited him, teaching him how to practice purposefully. Another participant agreed on the importance of college golf as an opportunity to gain experience and exposure. Several participants reiterated that experience significantly benefits golfers.

*Has given us a playing opportunity once a month*,* which is nice*,* but before that*,* yeah*,* it was the Sunshine Tour*,* which was six events*,* and then we wait around till August or December because LPGA’s Q-school is August and Ladies European Tour (L.E.T) in December*,* so you’d wait around for those times and go in with no tournaments played for six to eight months. (Participant 9)*

*And obviously did my four years there. I played for the university*,* played Division One*,* pretty top-level golf*,* and played against all the guards like Spieth*,* Schaeffler*,* and all those guys you see now. So*,* it was a really good experience that I had there. (Participant 4)*

*Because I’ve experienced that I’ve got playing on the Challenge Tour*,* those first two or three years*,* obviously has helped me quite a bit. (Participant 4)*

*And you’re gonna go through your struggles*,* and I think if you haven’t experienced those things early on*,* it can be difficult to handle when you’ve had so much success early on. (Participant 17)*

Hayman, Polman, Taylor, Hemmings and Borkoles [72] assert that golfers derive greater benefits from tournament experience than from spending numerous hours practising. This is evident from the average age of 35 years of PGA Tour golfers [110]. Moreover, Callan and Thomas [111] concluded that professional golfers’ earnings increase with the number of tournaments they participate in, underscoring the importance of experience in their career success. This relationship between tournament participation and financial success suggests that managing external pressures, such as financial concerns, could significantly affect performance.

#### Financial resources

The fifth theme derived from the data was financial resources, emphasising the importance of having adequate financial support during the development of professional golfers. Several participants underscored the significance of financial support during their professional development. Furthermore, players have highlighted that competing without the financial pressure to perform, positively affects their performance. A summary of the theme representing the relevance of financial resources is indicated in Table 6.

**Table 6:** Summary of the theme representing the relevance of *Financial resources*

Theme	Sub-themes	Theme description	Sample response
Financial resources	Financial pressure	Reduced financial pressure enhances performance.	*“So*,* I struggled to play top golf*,* paying back money having a sponsor being under that financial pressure.” (P1)* *“You don’t want to be thinking about money on the golf course.” (P15)*
	Financial support	Gaining financial support through sponsorships is essential.	*“Financial definitely you need to have money you need to have someone supporting you or you have to come from a really wealthy background.” (P3)* *“…and of course*,* money plays a big part in that because if you’re an amateur*,* don’t have money to go and play tournaments*,* then you don’t get that tournament experience*,* so it’s just a lot to do with financial things…” (P13)*
	Personal branding	Maintaining a professional image and an active social media presence is crucial, as strong performances and a polished image contribute to building a personal brand and attracting sponsors.	*“Definitely*,* I think personally*,* as a golfer*,* you are basically your company. That’s how I would see it. You are your own company*,* and you have to try to do half of that to expand yourself*,* advertise*,* and do that type of stuff.” (P3)* *“But I think branding yourself is important. I think if you can come across as a likeable character*,* of course*,* you’ve got to be yourself.” (P11)*

##### Financial pressure

Participants indicated that they perform better when not under financial pressure. They gain a sense of freedom when not concerned about the consequences of suboptimal performance. The financial pressure that participants experienced was detrimental to their performance. One of the players mentioned that despite having the financial support to compete in various events, the funds were only a loan, which meant that she played under financial pressure as she had to perform to earn enough money to repay the loan. Various participants agreed that playing without financial pressure is crucial.

*There’s gonna be years when you differ a little bit*,* and then that financial stress can make or break your career as income. (Participant 7)*

*He plays with freedom. He’s not trying to make money that week. He’s like*,* it’s not*,* it’s not about that he just wants to win the tournament. So*,* it’s like he’s gonna play*,* and he’s playing with complete freedom. Because I don’t think there’s any financial thing. (Participant 5)*

Consistent with the participants’ views, the professional development of golfers entails significant expenses, necessitating support from sponsors to increase the prospects of success while alleviating potential financial pressure [112]. Farrally, Cochran, Crews, Hurdzan, Price, Snow and Thomas [113] noted that many players struggle with substantial financial pressure even at the professional golfing level. The costs associated with coaching, equipment, and tournament travel can significantly influence an athlete’s developmental experience. When players receive adequate financial support, they are more likely to compete with a sense of psychological freedom, unburdened by underlying financial pressures [10].

##### Financial support

Many participants emphasised the importance of financial support for their success, asserting that sponsorships to cover various expenses are essential to becoming a professional golfer. One participant noted that his sponsor is a supporter and friend, highlighting the crucial role this relationship plays in his journey.

*Yeah*,* every single week*,* it was like*,* if I miss this pattern*,* I’m borrowing more money. How am I going to pay next week? How am I going to pay for it? And I was only playing 11 events? So*,* in terms of the bigger picture*,* it actually wasn’t a lot of money. But for somebody who was borrowing money*,* it was it was a lot. (Participant 7)*

*But if you know*,* it’s if your parents aren’t well-off*,* and you don’t have the money yourself*,* you’re going to find it very hard to continue to play and travel without pressure. (Participant 15)*

*Absolutely*,* I mean*,* the amount of travelling you do*,* especially in Europe*,* are so expensive. You know*,* it just helps immensely to go up*,* ladies. It’s no use getting your card and not being able to travel if you don’t have the finances*,* so at least they’ve given me the opportunity to go and do what I need to do. (Participant 12)*

*It’s more of a friendly relationship*,* and if you need him*,* you can call him. That type of stuff. So*,* I would say it’s more that he’s a sponsor*,* but that’s part of a team. It’s more a team than a sponsor*,* and it made a very big difference for me. (Participant 14)*

The professional development of golfers requires a large financial commitment, as stated by various participants, which may include coaching and travel expenses [71]. Fry, Bloyce, and Pritchard [26] and Fry and Bloyce [25] agree and emphasise the significant expenses that professional golfers have, and if they are to make golf a career, they must be successful for an extended period. Additionally, Portenga [71] highlights that golf is more costly than other sports because golfers require regular access to a golf course, necessitating paying golf course fees. However, when golfers obtain sponsorships that help them develop their personal brand, they significantly minimise the financial pressure and enhance their chances of success [112].

##### Personal branding

Linking personal branding with financial support, numerous participants expressed the importance of seeing themselves as a brand to gain sponsorships. Participants indicated that players should perform well on the golf course, be presentable, have good people skills and be able to network. Participants emphasised the importance of developing and maintaining a personal brand for golfers to secure financial support. Two golfers noted that while they have established personal brands, their on-course performance is their most effective marketing tool for enhancing their brand and attracting sponsorships. Others highlighted that a presentable appearance is essential for a successful brand. It was also noted that golfers need strong interpersonal skills, effective networking, and proper brand representation. One participant further emphasised the necessity for golfers to remain authentic to their identity.

*I mean*,* you have to always put yourself out there*,* whether it’s*,* you know*,* being on TV if needed*,* push as much as you can*,* so people see who you are*,* and become interested in*,* in your brand and your line of work. (Participant 3)*

*Yes*,* we’ve had to learn quickly how to actually even speak to potential sponsors. (Participant 8)*

*I’ve always been I’ve always been like*,* if I’m playing more*,* then*,* like*,* the sponsors would come because I don’t need to go on to social media to promote myself 30 posts a month and track it*,* you know what I mean*,* like*,* if I’m*,* if I’m doing well*,* then people will see that*,* you know. (Participant 4)*

*I think having a platform for that and then playing well with the eyeballs on you is going to be the best form of marketing. (Participant 11)*

*You need to network as well as you can*,* represent your brand. (Participant 7)*

As stated by the participants and found in the research of Fry, Bloyce, and Pritchard [26], professional golfers require a well-defined personal brand to secure sponsorships, which helps alleviate the financial challenges of pursuing a professional golf career. Roos and Muller [112] observed that securing sponsorships has a notably positive effect on the success trajectory of professional golfers. Sponsorships for equipment, apparel, and travel substantially alleviate financial burdens for golfers once attained [23]. Furthermore, to obtain a successful personal brand, golfers must consistently perform at a high level and have an enhanced physical appearance [112]. Consequently, players are encouraged to maintain their physical appearance with conditioning training, enhancing their personal brand [112] and improving performance [20].

#### Physical conditioning

The sixth theme that was constructed from the data was physical conditioning, highlighting its critical importance for personal branding and injury prevention, fatigue management, and the overall performance of professional golfers. Several participants indicated that physical training and warm-up are crucial, assisting them in concentrating, gaining clubhead speed, avoiding injuries, and decreasing fatigue levels after a golf round, thereby allowing golfers to practice more effectively after tournament competitions. A summary of the theme representing the relevance of physical conditioning is indicated in Table 7 below:

**Table 7:** Summary of the theme representing the importance of *Physical conditioning*

Themes	Sub-theme	Theme description	Sample response
Physical conditioning	Warm-up	Assist with injury prevention and mobility improvement through stretching and dynamic exercises.	*“I’ve just made sure that my body is in a good place. I’ve activated all the right muscles and then warmed up correctly.” (P17)* *“So*,* I’m going to try to see the physio as much as possible*,* and then I’ll try*,* but then I’ll just do the stretching exercises every day to loosen up and so on.” (P13)*
	Injuries	Physical conditioning is essential to prevent injuries.	*“Yes. Yeah*,* I do. Trying obviously…prevent injury*,* stay flexible*,* all of those good things.” (P2)* *“Injury prevention first thing to not get injured.” (P8)*
	Fatigue levels	Maintaining adequate physical conditioning assists with fatigue during and after competition.	*“The whole 12*,* 13*,* 14 stretch is a lot easier than it used to be kind of thing. Just from even just concentrating you know*,* due to fatigue*,* mentally. Even just being in the gym helps avoid that.” “I also find now if I have to hit balls after a round*,* I can.” (P8)* *“…but for me*,* the most important thing is that after 18 holes I am no longer so stiff and sore and tired…” (P6)*
	Performance enhancement	Enhanced clubhead and ball speed through strength training and conditioning.	*“Yes. Yeah*,* I do. Trying obviously*,* gaining speed…” (P2)* *“For me is speed. I’m not the longest hitter just to try and get the clubhead speed up.” (P8)*

##### Warm-up

Several participants emphasised the importance of incorporating warm-up and stretching exercises into their tournament preparations, often with the assistance of a physiotherapist. They highlighted that a thorough warm-up is essential for optimal performance.

*I’ve just made sure that my body is in a good place. I’ve activated all the right muscles and then warmed up correctly. (Participant 17)*

Implementing comprehensive sports science methodologies, including tailored strength and conditioning programmes, as well as structured warm-up and cool-down routines specifically designed for golfers, has been demonstrated to significantly enhance performance and potentially mitigate the occurrence of injuries in the sport [30].

##### Injuries

Several participants stated that their primary reason for engaging in physical conditioning is to prevent injuries.

*So*,* physical condition is so important for preventing injury*,* more so than anything. (Participant 5)*

Lee [23] highlighted that physical factors, particularly injuries, are significant contributors to the discontinuation of participation in golf. Physical conditioning plays a critical role in mitigating these issues, as improved fitness levels can lead to a reduced occurrence of injuries [114]. When athletes experience physical fatigue, their motor actions diminish, adversely affecting their performance [115]. Consequently, maintaining optimal physical conditioning is essential not only for enhancing performance but also for ensuring longevity in the sport by minimising injury risk and effectively managing fatigue.

##### Fatigue level

One player reported that physical training enhances concentration during the concluding holes of tournament play and supports the post-round practice. Additionally, participants noted that physical training and collaboration with a physiotherapist help reduce fatigue.

*The most important thing is that after 18 holes*,* I am no longer so stiff and sore and tired. (Participant 6)*

As noted by participants and found by Coughlan, Taylor, Wayland, Brooks, and Jackson [20], maintaining a high fitness level as a professional golfer can help preserve clubhead speed and ball speed, preventing the declines often associated with the off-season. According to Coyne [29], maintaining good cardiovascular fitness is essential for sustaining energy and focus throughout the round, thereby minimising the likelihood of fatigue-induced errors. This high-level fitness training regimen allows athletes to maintain peak performance levels, which may otherwise diminish without consistent physical conditioning and enhanced fitness [20]. Consequently, physical conditioning could enhance performance [116].

##### Performance enhancement

Several participants emphasised that physical conditioning enhances clubhead speed and contributes significantly to overall performance improvement.

*Because he’ll see your body*,* your body’s not moving so well*,* you need to go work with so and so in the gym*,* work it out*,* stretch*,* come back*,* and we’ll see*,* you know if it’s any better. (Participant 7)*

*So*,* I’ve been doing TPI for the last two years*,* and my body’s grown*,* I’ve become stronger*,* hitting the ball further. More stable in my golf swing*,* don’t really make many bad swings anymore. (Participant 15)*

Yoon, Kim, Kwon, Ahn, Kim, and Kim’s [104] findings correspond with the participants’ responses as they found that adding strength and fitness improves golfers’ performances, while Coughlan, Taylor, Wayland, Brooks, and Jackson [20] also indicate that physical conditioning increases golfers’ clubhead speed. Furthermore, this physical conditioning should become a habit for golfers and form part of their lifestyle [117].

#### Lifestyle habits

The seventh theme derived from the semi-structured interviews was lifestyle habits, underscoring the professional lifestyle that golfers must adopt. It also drew attention to the fact that this developmental process entails numerous sacrifices. Participants stressed the potential loneliness of the professional golfer’s lifestyle and emphasised the importance of surrounding oneself with the right individuals. Additionally, many participants highlighted that maintaining a disciplined lifestyle was crucial to success. A summary of the theme representing a golfer’s lifestyle habits is indicated in Table 8.

**Table 8:** Summary of the theme representing a golfer’s *Lifestyle habits*

Themes	Sub-theme	Theme description	Sample response
Lifestyle habits	Lifestyle needed for success	Includes discipline, sacrifices, managing loneliness, and coping strategies for poor performances.	*“I need to know that everything around me is in symbiosis for me to perform at my best*,* and having a family*,* husband*,* a home*,* a job.” (P1)* *“Negative stuff*,* it’s not negative*,* but it’s challenging like we have now. I travelled abroad in Europe last year*,* and nobody really understands you. Driving on the other side of the road makes it a bit difficult to find your feet*,* and sometimes*,* it’s difficult to live out of a suitcase. It’s hard to go to a different place every week. Yes*,* you’re never really at home. It’s a bit difficult sometimes.” (P6)*
	Self-care, nutrition, and sleep	Healthy eating, adequate sleep, and health monitoring with technology like Whoop are essential for optimal performance. Continuous carbohydrate intake and sufficient rest are crucial for maintaining concentration and overall effectiveness.	*“…doing everything they can*,* eating well*,* sleeping well…” (P12)* *“…you know*,* sleeping*,* well*,* eating well*,* that sort of thing.” (P17)*

##### Lifestyle required for success

Various participants specified that a professional golfer needs a disciplined lifestyle, and golfers must make sacrifices in multiple areas of their lives. Furthermore, participants said that golf is a lonely lifestyle, and golfers must surround themselves with the correct people. In addition, how golfers behave after subpar performances is crucial. Several participants emphasised the need for a balanced lifestyle, indicating that achieving this can enhance tournament performance. One participant described a significant lifestyle change, specifically abstaining from alcohol, which has had a notably positive impact. Others noted that the lifestyle of a professional golfer could be selfish and lonely, while the challenges of travelling and living out of a suitcase were also highlighted.

*But then*,* yeah*,* I think you’ve kind of got to be doing everything you can away from the course in terms of training*,* sleeping well*,* eating well*,* that sort of thing. To find success on the course*,* I think*,* what I’ve learned it’s not about standing on the range for 10 h. And it’s not about playing a round of golf every day. It’s more if I’m doing the things off the course well*,* I can trust that once I get to the golf course*,* I’m gonna be in pretty good shape to play some decent golf. (Participant 17)*

*You know*,* I’ve got to sometimes put golf before a lot of things*,* obviously not before family*,* but it’s definitely*,* it’s a selfish life. And just can be very lonely*,* travelling and being out there by yourself*,* which you have to manage. (Participant 3)*

One participant noted that travelling to various locations with fellow competitors can enhance the golfers’ experience. Another participant deemed sacrifices essential for those pursuing a professional career, while yet another emphasised that the lifestyle of a professional golfer varies significantly among individuals. Additionally, the importance of golfers’ behaviours following subpar performances was highlighted, with a focus on how top-quality golfers prioritise immediate practice sessions after such experiences.

*In the last half of the season*,* I have started travelling with another guy*,* and that makes it much better. (Participant 13)*

*So*,* there has to be a certain amount of sacrifice for you to make it as any kind of professional athlete. You can’t have both. (Participant 7)*

*Now that’s the difference between*,* say*,* the normal guys*,* is the difference is when things go bad*,* you’re still going to train with the intensity that you’re going to do well next week. (Participant 16)*

Professional golf is often portrayed as a glamorous career path that offers substantial financial rewards and minimal concerns [25]. However, the reality is that a small number of professional athletes earn a significant financial income. Professional golfers require great commitment, demanding a significant lifestyle change [118, 119]. Transitioning from a laid-back lifestyle, individuals must adjust their routines to prioritise rest while mentally preparing for the next day’s training. Additionally, the travel involved necessitates long periods away from loved ones and living out of a suitcase, often in basic accommodation while managing finances [118]. Moreover, professional golfers often experience feelings of loneliness, struggle to maintain a balanced work-life approach and encounter challenges in their social relationships [22, 25]. As such, golfers should be aware of the mental health challenges that may surface when attempting a career in professional golf [120, 121]. Inadequate self-care, including insufficient sleep and nutrition, can also adversely affect athletes’ mental performance and pose further challenges to their overall performance [117].

##### Self-care, nutrition and sleep

Participants indicated that golfers must take care of all aspects of their lives, including their health, sleep, and diet. Adequate sleep and nutrition were mentioned as crucial factors that enhance performance during tournaments. Numerous participants use wearable technology such as Whoop to gain insights into their bodies’ responses to stressors and activities, allowing them to optimise training and overall well-being.

*Whether you’re talking about discipline in terms of diet or sleep. (Participant 11)*

*I would say it is 30% mental*,* 30% technical*,* 30% course management*,* and the other 10%*,* I would say probably physical and eating. (Participant 16)*

*I use the whoop. And literally*,* I tested it. (Participant 15)*

In line with the participant’s responses, golfers should prioritise comprehensive self-attention across various aspects of their lives, including mental health [120], nutrition [122, 123], and sleep [124]. More specifically, Nagashima, Ehara, Ehara, Mitsume, Kubo and Mineo [122] explain that the nutrition of golfers significantly impacts their energy levels, highlighting the importance of mindful food intake during a round of golf to sustain energy levels and enhance performance. Furthermore, golfers require adequate sleep for optimal human functioning and performance [125]. In addition, inadequate sleep can negatively impact a golfer’s psychological performance [117].

#### Psychological proficiency and talent

The eighth theme derived from the semi-structured interviews was the importance of psychological proficiency and talent, which underscored the importance of mental skills, personal identity, work ethic, determination, and talent. Participants underscored the critical role of confidence and self-belief in achieving success, urging golfers to trust their abilities. They emphasised the inevitability of negative thoughts and stressed the importance of mental toughness in effectively managing them. Additionally, participants highlighted the necessity for golfers to commit to success while maintaining realistic expectations. Furthermore, participants consistently stressed the critical importance of a robust work ethic and perseverance to succeed, emphasising that golfers must have the discipline and drive to succeed. However, these psychological skills must be complemented by a certain level of natural talent. A summary of the theme representing a golfer’s psychological proficiency and talent is indicated in Table 9.

**Table 9:** Summary of the theme representing a golfer’s *psychological proficiency and talent*

Theme	Sub-themes	Theme description	Sample response
Psychological proficiency & talent	Mental skills	Developing confidence, focus, and mental toughness to manage negative thoughts and pressure effectively.	*“I mean*,* you could say attitude or*,* or just mental approach.” (P2)* *“I think obviously*,* the mental stability and mental toughness is key.” (P12)*
	Fostering personal identity	Enable players to develop a distinct identity and to avoid imitation or dependence on external validation.	*“…and now you are just a number to sort of try and establish your own identity is a big thing.” (P1)* *“So*,* the thing is you*,* you are an individual. So you have to work on what you do and around you. It’s an individual sport*,* and the reality is*,* unfortunately*,* that’s why it’s difficult because the actual recipe for success is to learn who you are as a person and how you do well…” (P16)*
	Work ethic	Refers to a player’s dedication, discipline, and commitment to consistently improving their skills and performance.	*“And like what I thought working hard was wrong*,* these guys showed me what working hard was.” (P4)* *“…it’s hard*,* it’s hard work*,* and endurance is the best…” (P13)*
	Determination and perseverance	This refers to strong motivation, perseverance, and consistent commitment while maintaining a positive attitude, further enhancing their ability to excel.	*“As a finished product*,* someone who has to have a lot of grit and a lot of determination*,* and a work ethic that has to be second to none*,* really.” (P11)* *“Having the drive to be number one*,* not just playing to make money*,* you want to drive to be number one*,* or you want to be*,* you want to drive to win. Those are the guys who are successful.” (P5)*
	Talent	Success in golf requires a certain physiological proficiency, including talent and innate capability, to achieve one’s goals.	*“And then you also have to have a certain amount of talent…” (P7)* *“Well*,* I would say*,* first of all*,* you have to have the talent for it.” (P14)*

##### Mental skills

Several participants indicated that confidence and self-belief are crucial to success and that they should trust their abilities. Furthermore, participants underlined the fact that golfers should realise that negative thoughts will occur and that dealing with them through mental toughness is essential. Participants stated that golfers should commit to success and have realistic expectations. Several participants accentuated the mental aspect of golf as central to success. One noted that maintaining daily mental focus is crucial for enhancing performance, while another reflected on early career challenges related to emotional immaturity and the need for support. Additionally, they stressed the acceptance of negative thoughts and effective management of such emotions as a critical component of mental resilience in the sport.

*Yes*,* he*,* that’s his focus. It is a lot of daily mental focus and that type of stuff*,* like staying more focused to notice what you’re thinking. (Participant 13)*

*I was very much emotionally immature. Mentally immature in terms of I didn’t actually know how to play golf. (Participant 7)*

Self-belief and confidence are essential ingredients for golfers, emphasised alongside focus and discipline. One participant noted the strategy of slowing down in pressure situations, opting to handle challenges independently rather than relying on a sports psychologist. Another preferred way is discussing experiences with fellow athletes rather than receiving psychological counselling. Participants also stressed trusting one’s talent and accepting the journey’s difficulties. It was noted that early career success can lead some players to neglect their process and work ethic, with one participant explicitly stating that such success often results in reduced effort and a departure from established routines. The importance of confidence, commitment, and realistic expectations was underscored, along with a warning that golfers frequently set their expectations too high and must maintain the correct mindset to succeed.

*It’s definitely a mental state*,* but just knowing that you belong*,* you know*,* like being there like*,* I can almost have that attitude that no matter what game I’ve got on the day*,* I can still beat you guys*,* that air of confidence. (Participant 3)*

*A lack of self-belief*,* it’s easy to fall into that trap. Yeah*,* I think that’s*,* that’s the biggest problem I see is that lack of focus on what you want to do like a bigger picture*,* and lack of discipline. (Participant 11)*

*So*,* to hear it coming from somebody who’s actually been in those positions*,* I think*,* is way more useful than somebody who studied sports psychology. (Participant 7)*

*You know*,* like*,* a lot of youngsters*,* the expectations are way too high. They don’t realise that golf is bloody difficult. (Participant 17)*

*Number two*,* mindset. If you have a weak mindset*,* you’ll never make it. You can be the most talented golfer in the world. But if you don’t believe in yourself*,* that you*,* that you can beat everyone*,* then you never will. So*,* it’s all about the mindset. (Participant 15)*

*And then*,* especially if you have success*,* you think that it’s going to last forever. So*,* they back off of the process that they’ve had and that they’ve done to get to that point. But it’s actually once you’ve made it*,* it’s when you have to put it in. That’s when the real work starts because you have to be able to stay there. (Participant 7)*

MacKenzie [126] agrees with the participants’ views and emphasises a robust psychological skill set such as focus and concentration, mindfulness and being present, emotional regulation, confidence, positive attitude, mental visualisation, and goal setting. According to Hayman, Borkoles, Taylor, Hemmings and Polman [70], investing in mental skills training becomes crucial as golfers spend more time in deliberate practice. Maturity and proficient psychological skills are essential, as golfers often confront challenges and setbacks during their performances. In a study by Mattsson, Hassmén, McCullick and Schempp [73], Swedish golfers indicated that psychological factors outweigh physiological factors when developing golfers. According to Aparicio, Fried, Pastor and Tauer [18], professional golfers frequently encounter high-pressure situations where previous experiences can positively affect their competitive performance. When golfers evaluate their performance, make the required adjustments, and foster a solid personal identity by maintaining their natural ability, they benefit significantly from enhanced resilience and consistency in their game [38, 44].

##### Fostering personal identity

Participants underlined the importance of golfers recognising the value of cultivating their own identity rather than imitating other professionals. They highlighted the need to adopt strategies and techniques that align with individual strengths and preferences and seek external approval.

*And this year has been a big one for me in terms of understanding myself as a player and understanding my game and understanding what I’m really capable of and trusting that and being able to do it*,* you know? (Participant 17)*

*And I think if you are pressured by people that you seek approval from*,* you are destined to be*,* you know*,* upper pathway*,* that has a very fine margin for error. (Participant 11)*

Participants stated that golfers frequently alter their approach upon reaching a professional level, feeling compelled to mimic the swings of their peers, a sentiment that Roos [44] supports. This tendency can prove detrimental, as it may lead golfers to forsake the innate abilities that initially propelled them to elite status. Highfield [38] agrees, advocating for golfers to nurture their natural abilities rather than undergo radical transformations. Therefore, golfers are encouraged to remain authentic to their identities. In addition, a robust work ethic is essential for golfers to achieve success at the highest level [70].

##### Work ethic

Participants underscored that a lack of solid work ethic severely hinders success. They recommended that developing confidence, determination, and a focused approach to objectives are vital.

*I said he hasn’t got the most talent. Reasonably talented. Fantastic work ethic. (Participant 5)*

*And then you also have to have a certain amount of talent and work ethic for everything to come together. (Participant 7)*

Participants mentioned work ethics as a vital factor, a view that coincides with that Laumakis [127] and Portenga [71]. Regarding professional golfers’ work ethic, few would argue that Ben Hogan belongs on the list. Many consider Hogan to be one of the most hard-working golfers of all time. His unwavering dedication to progress cemented his place among the greatest golfers of all time [127]. Therefore, to attain elite status, golfers must invest significant time and effort in their development [70–72, 128, 129]. Throughout their professional development, golfers will encounter setbacks, necessitating a high level of self-determination and perseverance to achieve success [130].

##### Determination and perseverance

To be successful, golfers must have a high degree of achievement behaviour characterised by perseverance, persistent devotion, and a growth mindset. Participants emphasised that motivation is essential for success, with perseverance identified as vital for professional golfers. The need for a consistent commitment to excellence was highlighted, as was the importance of maintaining the correct attitude.

*I think it’s just like perseverance. (Participant 9)*

*That consistency and commitment and good is a lot*,* and consistency is actually a bigger factor. (Participant 14)*

In line with Rotella [91], a golfer’s commitment and time dedicated to self-improvement are crucial for success. Psychological commitment plays a material role in shaping behavioural outcomes that affect individual motivation. Factors such as sustained task engagement, the intensity of effort exerted, and the resilience to overcome setbacks in pursuing success are essential [130]. Additionally, Scanlan, Carpenter, Simons, Schmidt, and Keeler [92] propose five factors that characterise sports commitment: enjoyment derived from participation in the sport, personal investment in the activity, social constraints affecting involvement, alternative opportunities for engagement, and available opportunities for participation. In addition to golfers’ commitment, their proficiency to master and execute specific golfing skills under tournament conditions is also vital [18].

##### Talent

Several participants noted that while a strong work ethic and perseverance are essential for players, a certain level of talent is also necessary. Participants indicated that golfers require a certain level of talent and innate ability to achieve success.

*Well*,* I think it’s a skill level. I think being a professional golfer is an absolute package of a whole lot of things*,* it is skill*,* talent*,* work ethic*,* having the drive to be number one. (Participant 5)*

*Well*,* I would first say that you need to have the talent for it; you need to have the ability. If you don’t have that*,* hard work will take you somewhere*,* but if you lack the talent*,* it won’t help. (Participant 14)*

According to participants’ responses and the work of Brožka, Carson, Komarc, Zahálka and Gryc [131], players must have a particular skills level during development to successfully transition from amateur to professional level. A range of specific motor skills shapes golf performance, each associated with different shots, such as putting, chipping, short-range and long-range approach shots, and tee shots [131]. Apart from the specific motor skills needed, players who achieve elite status put in substantial time and investment in their development [71]. Therefore, a golfer’s inherent ability to master and execute specific golfing skills under tournament conditions is central [18]. Moreover, golfers must implement comprehensive course management strategies during tournaments [132].

#### Course management

The ninth theme elucidated from the semi-structured interviews was course management, emphasising the profound effect of strategic planning, shot selection, and data management systems such as Decade Golf on the performance of professional golfers. Several participants highlighted that using course management tactics through Decade Golf is crucial for golfers’ success. Participants indicated that golfers should understand their strengths and weaknesses to optimise performance. Furthermore, participants emphasised that good course management assists golfers in minimising risks, avoiding hazards, and strategically positioning themselves for favourable shots, ultimately leading to lower scores. A summary of the theme representing the importance of course management is indicated in Table 10.

**Table 10:** Summary of the theme representing the importance of *Course management*

Themes	Sub-theme	Theme description	Sample response
Course management	Strategic planning	Employing strategies such as Decade Golf, optimising shot selection, minimising bogeys and enhancing overall performance.	*“And then*,* you know*,* plotting your way around the golf course in a smart and strategic way allows you to free up. The way I see it*,* you know*,* like*,* if you know that you’ve got 65 yards between your target*,* you know you’re going to have to hit a really bad one to miss to be out of good position.” (P17)* *“I’m not striking the ball well*,* but I’m just managing myself around the course so well with the strategy.” (P15)*
	Shot selection	Anticipating worst-case scenarios in shot selection.	*“I’m just managing myself around the course so well with the strategy.” “I’ve realised that you can’t try and make more birdies; you needed to try and make less bogeys.” (P15)* *“not forcing birdies and minimising the bogeys and the mistakes.” (P17)*
	Decade Golf	Minimising errors and shot dispersion patterns and enhancing performance on par fives and short iron shots.	*“Its been the absolute best thing for my game*,* the absolute best thing for my game. I think I’ve had I’ve had three ball-striking weeks where I thought*,* okay*,* geez*,* I actually really ball-striked it this week*,* hardly missed a shot. And I went two wins and a second. And that’s since I started using Decade.” (P15)* *“Big*,* big. Yeah. I’ve used Decade Golf since I was in college. And I think that’s been instrumental in helping me understand how to play professional golf.” (P17)*

##### Strategic planning

Participants underscored the importance of a strategic approach in selecting appropriate clubs for shots, targeting strategic landing zones to optimise subsequent plays, and evaluating risk versus reward when deciding between conservative and aggressive strategies. Participants indicated that navigating themselves around the golf course strategically is essential.

*Plotting your way around the golf course in a smart and strategic way allows you to free up. The way I see it*,* you know*,* like*,* if you know that you’ve got 65 yards between your target*,* you know you’re going to have to hit a really bad one to miss to be out of good position. (Participant 17)*

Various participants use the strategic approach suggested by Decade Golf, which involves a method to evaluate the final position of the golf ball beyond mere proximity to the hole. This method relies on the research conducted by Mark Broadie [132]. He is known for pioneering the “Strokes Gained” methodology, which systematically assesses the effectiveness of each golf shot by analysing comprehensive data on the performance of professional golfers. This method has become widely recognised for its ability to quantify the impact of individual shots on overall performance and scoring in golf. The process offers a robust framework to assign a numerical value to each possible shot outcome. It calculates the difference in expected shots to finish the hole between the starting and ending positions of the ball. For instance, transitioning the ball from 180 yards on the fairway to eight feet from the hole yields a gain of 0.58 strokes. This improvement reflects a reduction of 1.58 strokes in position (from an expected 3.08 shots to 1.50 shots to finish the hole), achieved with a single shot. Therefore, this shot is appraised as generating 0.58 strokes gained [132]. Consequently, professional golfers prioritise shots that offer the highest strokes gained value to optimise their performance.

##### Shot selection

According to the participants, effective strategic planning enhances scoring opportunities while reducing mistakes, thereby enhancing overall performance during play. As such, the participants stated that golfers should attempt to make fewer bogeys, not more birdies.

*Not forcing birdies and minimising the bogeys and the mistakes. (Participant 17)*

According to various participants and Scott Fawcett from Decade Golf, the decision making of golfers should embrace the possibility of the worst possible outcome of a golf shot [21]. Decade Golf’s research indicates that even golfers of the highest ability will probably not hit the ball exactly where they intend to. Assessing shot dispersion patterns, it was noted that right-handed golfers typically hit longer shots on the left and shorter shots on the right [132]. Consequently, many golfers use the Decade Golf strategic system to improve their performance on the course [21].

##### Decade golf

Participants underscored the significance of course management, affirming their use of the Decade Golf system and its notable performance enhancement.

30% course management. (Participant 16)

*I started Decade Golf. It’s been the best thing for my game. (Participant 15)*

According to Decade Golf [133] and the study’s participants, course management is critical for golfers looking to succeed. Scott Fawcett invented Decade Golf, a course management system that uses shot dispersion patterns and PGA Tour scoring statistics to make strategic decisions on the golf course [133]. This approach to golf strategy involves golfers evaluating shot dispersion and employing a methodical approach based on percentage-based shot selection to optimise their overall score [134]. According to Decade Golf [133], there are five crucial statistics that every golfer should monitor. The approach focuses on techniques to reduce errors, such as avoiding double bogeys, bogeys on par fives, three-putts, using more than two chips every hole, and preventing bogeys when using a nine iron or less within 140 m [21]. In the Decade Golf course management system, the primary focus is anticipating the worst possible outcome and strategising to minimise the likelihood of encountering it [132].

### Strengths and limitations

This study aimed to elucidate the key factors that contribute to the development of professional golfers in a South African context. A key strength of this study is that data were collected from a combined sample of prominent professional golfers (*N* = 15) in South Africa and PGASA teaching professionals (*N* = 2). This included five male, and five female professionals ranked among the top 30 on the Sunshine Tour order of merit, providing a rich and diverse dataset. The study was strengthened by ensuring a nearly equal representation of male and female professional golfers among the participants. Furthermore, the interviews were open-ended, allowing the participants to discuss any factors that may add value to the study. The researcher, who is a PGASA teaching professional with extensive experience, was well-positioned to ask pointed questions, thereby enhancing the depth and richness of the data collected for the study. Despite these strengths, a key limitation of the study is that data collection captured participants’ perspectives at a single point in their playing or coaching careers, limiting insight into how their views may evolve over time. A more in-depth understanding of the subject matter could have been achieved with a longitudinal approach, allowing for the documentation of a professional golfer’s entire career trajectory. Future research endeavours might consider using an ethnographic methodology to thoroughly examine the environments in which professional golfers operate over extended periods of time, thereby yielding additional insights on the subject. Moreover, intervention studies could be conducted within the professional golfing community, implementing the factors identified in this study and assessing their effects on the professional development of golfers.

### Conclusion

Through the lens of an ecological approach, this study identified key factors that influence the professional development of golfers. Themes such as ‘Supportive developmental environment’, ‘Career management’, ‘Team of professional staff’, ‘Lifestyle habits’, and ‘Psychological proficiency and talent’ align with Bronfenbrenner’s ecological model [54], highlighting the interplay of individual skills, support systems, and environmental factors in a golfer’s progression. Additional themes, including ‘Competition-specific training’, ‘Financial resources’, ‘Physical conditioning’, and ‘Course management’, further underscore the diverse elements critical to professional success.

The main pillars of any golfer’s development plan when pursuing professional play should be effective coaching, a supportive and pressure-free environment, and comprehensive career management, along with elements such as financial support, specialised competition training, physical and psychological preparedness, and a strong support system. Together, these elements provide a holistic understanding of the factors that contribute to professional golfers’ success. Moreover, this study presents valuable insights into the key factors that influence the professional development of golfers, offering a deeper understanding of the unique challenges and opportunities within the South African golfing landscape. By highlighting the specific needs and conditions that shape golfers’ professional development in South Africa, the findings lay the groundwork for developing a context-specific player development framework. This framework could serve as a tailored model for enhancing support across critical areas such as coaching, mental preparation and conditioning, and career advancement, ultimately helping to optimise the development pathways for professional golfers in the country.

## Electronic supplementary material

Below is the link to the electronic supplementary material.

Supplementary Material 1

Supplementary Material 2

## Data Availability

The data for this study was stored and made available in Open Science Framework and can be viewed at the following link: https://osf.io/6e3y5/?view_only=fc5f849d0f2e4c0691f2d29f1e5d0bfd.

## Abbreviations

ATDEM: The athletic talent development environment model
DMGT: The differentiated model of giftedness and talent
DMSP: The developmental model of sport participation
DP: Dubai Ports
FTEM: The foundations, talent, elite and mastery framework
HREC: Health Research Ethics Committee
LET: Ladies European Tour
LARPE: Life-span model of the acquisition and retention of perceptual-motor expertise
LTAD: Long-term athlete development 2.1
PGA: Professional Golfers Association
PGASA: Professional Golfers Association of South Africa
SAGA: South African Golf Association
SAKG: South Africa Kids Golf
USA: United States of America
USKGF: The United States Kids Golf Foundation
WGSA: Women’s Golf South Africa
