# Supplementary material for: Key factors influencing the professional development of golfers in South Africa: a reflexive thematic analysis
Source: BMC Sports Sci Med Rehabil. 2025 Jul 16;17:205. doi: 10.1186/s13102-025-01239-7 (PMC12269114; doi:10.1186/s13102-025-01239-7)
Supplement: Supplementary file 1 — Supplementary Material 1 [file 13102_2025_1239_MOESM1_ESM.docx]

**DEMOGRAPHIC SHEET for research participants**

| 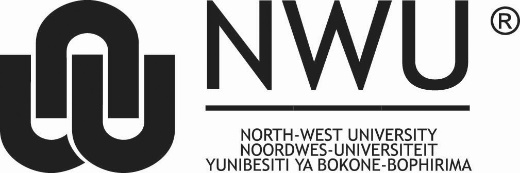 | Private Bag X1290, Potchefstroom South Africa 2520  Tel: +2718 299-1111/2222 Fax: +2718 299-4910 Web: http://www.nwu.ac.za |
| --- | --- |

**TITLE OF THE RESEARCH STUDY: Evaluating the feasibility of a professional golfer’s development framework within the South African context**

**Please complete the following questions:**

| 1. What is your name and surname? |  | | | |
| --- | --- | --- | --- | --- |
| 1. In which country do you reside? |  | | | |
| 1. What is your e-mail address? |  | | | |
| 1. What is your contact number? |  | | | |
| 1. What gender are you? | Male | Female | | Other |
| 1. What is your language preference? (Mark with X) | English | | Afrikaans | |
| 1. Are you an amateur or professional golfer? |  | | | |
| 1. If you are a professional golfer, on which professional tour do you compete on? |  | | | |
| 1. For how long have you been competing on a professional tour? |  | | | |
| 1. What is your current amateur or professional ranking? |  | | | |
| 1. What is your highest achievement as a player? |  | | | |
| 1. At what age did you start playing golf? |  | | | |
| 1. Would you like to receive a copy of the research study once completed? |  | | | |
