# Supplementary material for: Key factors influencing the professional development of golfers in South Africa: a reflexive thematic analysis
Source: BMC Sports Sci Med Rehabil. 2025 Jul 16;17:205. doi: 10.1186/s13102-025-01239-7 (PMC12269114; doi:10.1186/s13102-025-01239-7)
Supplement: Supplementary file 2 — Supplementary Material 2 [file 13102_2025_1239_MOESM2_ESM.docx]

**INTERVIEW SCHEDULE 1**

(Current South African Sunshine Tour, Asian tour, European and PGA tour professionals and amateurs in the top 100 rankings).

**Introduction**

Research has indicated that various aspects contribute to the transition of professional golf players to senior elite status. The professional golfer development framework (PGDF) ([Roos, 2018:92](#_ENREF_47)) provided certain themes that may expand the understanding of key factors in this transition process. This research study aims to identify shortcomings in the existing PGDF and explore further aspects that may assist golf players’ development at different levels of participation. The themes provided in the PGDF and others as identified from literature forms the basis of the interview questions and will be contextualised according to the particular participant:

- In as much detail as possible, please describe the journey your career has followed from a young age up to the present moment?
- According to you, what are possible constraints that may hinder the process of becoming a professional golfer?
- Looking back over your career, which aspect made the largest impact on you in terms of your professional development in golf? Both positive and negative.
- Identify and describe the factors that have contributed to your performance and professional development in golf?
- Explain which aspects would you regard as essential to transfer from amateur to professional golf?
- Identify and describe the characteristics that you believe the elite (ranked in the top 10) golf professionals have that the less elite don’t have?
- Are there any other aspects that you feel play an important role in the development of a golf player and that we might have missed?

The following probes will be used to gain more information:

- Why do you say that?
- Could you provide us with more detail/examples?

Thank you very much for taking the time to complete this interview. It is much appreciated.
